# Anodic Electrogenerated Chemiluminescence of Ru(bpy)_3_^2+^ with CdSe Quantum Dots as Coreactant and Its Application in Quantitative Detection of DNA

**DOI:** 10.1038/srep15392

**Published:** 2015-10-16

**Authors:** Yong-Ping Dong, Ting-Ting Gao, Ying Zhou, Li-Ping Jiang, Jun-Jie Zhu

**Affiliations:** 1 State Key Laboratory of Analytical Chemistry for Life Science, School of Chemistry and Chemical Engineering, Collaborative Innovation Center of Chemistry for Life Sciences, Nanjing University, Nanjing 210093, China; 2 School of Chemistry and Chemical Engineering, Anhui University of Technology, Maanshan 243002, China

## Abstract

In the present paper, we report that CdSe quantum dots (QDs) can act as the coreactant of Ru(bpy)_3_^2+^ electrogenerated chemiluminescence (ECL) in neutral condition. Strong anodic ECL signal was observed at ~1.10 V at CdSe QDs modified glassy carbon electrode (CdSe/GCE), which might be mainly attributed to the apparent electrocatalytic effect of QDs on the oxidation of Ru(bpy)_3_^2+^. Ru(bpy)_3_^2+^ can be intercalated into the loop of hairpin DNA through the electrostatic interaction to fabricate a probe. When the probe was bound to the CdSe QDs modified on the GCE, the intense ECL signal was obtained. The more Ru(bpy)_3_^2+^ can be intercalated when DNA loop has larger diameter and the stronger ECL signal can be observed. The loop of hairpin DNA can be opened in the presence of target DNA to release the immobilized Ru(bpy)_3_^2+^, which can result in the decrease of ECL signal. The decreased ECL signal varied linearly with the concentration of target DNA, which showed the ECL biosensor can be used in the sensitive detection of DNA. The proposed ECL biosensor showed an excellent performance with high specificity, wide linear range and low detection limit.

Electrogenerated chemiluminescence (ECL) is a light emission that arises from the high-energy electron-transfer reaction between electrogenerated species. ECL is attracting more attention due to its low cost, wide linear range, simple instrumentation, and high sensitivity^1^. Since it was first observed in the early 1970s, the Ru(bpy)_3_^2+^ ECL has been extensively studied and widely used in immunoassays and DNA probe assays^2^,^3^,^4^,^5^,^6^,^7^,^8^,^9^. Coreactant is often needed in Ru(bpy)_3_^2+^ ECL and numerous analytes, such as oxalate, amino acids, and tri-n-propylamine (TPA), can serve as coreactants^10^,^11^,^12^. Among them, TPA is mostly used in Ru(bpy)_3_^2+^-based ECL biosensor because TPA can produce the highest light emission^4^,^13^,^14^. TPA radicals can be generated via direct electrode oxidation to react with Ru(bpy)_3_^3+^ to form [Ru(bpy)_3_^2+^]* which then decays to produce orange emission centered at 610 nm^15^. However, the oxidation of TPA on the electrode surface is not as fast as desired. Moreover, TPA is highly volatile, poisonous, and low solubility, which makes experimental operation relatively difficult, and this has driven the search for new coreactants of Ru(bpy)_3_^2+^. On the other hand, during the introduction of coreactants into the solution, the coreactant-related physicochemical behaviors like diffusion, absorption, as well as its concentration, may complicate the ECL systems. Because ECL reaction involves both electrochemical and chemical reactions, it is possible to find new coreactants using chemically modified electrode. For immunoassay and DNA analysis, new coreactants with high ECL efficiency, low ECL background and user-friendliness are desired^16^.

Due to the unique quantum size dependent electrochemical properties of QDs and controllable ECL merits, QDs ECL has become more and more fascinating^17^,^18^. ECL applications of QDs are almost based on the cathodic ECL in the presence of coreactants^19^,^20^,^21^,^22^,^23^. However, other electrochemical characters, such as electrocatalytic behavior of QDs, have not attracted much attention. It has been found during the investigation of anodic ECL of QDs that the cation radical QD^+•^ can be directly produced from the electro-oxidation of QDs^24^,^25^. Since the oxidization process of QDs is similar to that of TPA, it is reasonable to speculate that the QDs modified on the GCE might act as a coreactant to generate anodic ECL with Ru(bpy)_3_^3+^, which can sufficiently avoid the drawback of the introduction of coreactant in the solution and can find new roles of QDs in ECL investigation.

Nucleic acids have long been considered as a kind of molecule with genetic information. The development of sensitive and selective methods for the detection of trace amount of sequence specific DNA is of great importance in clinical diagnosis, food analysis, and environmental monitoring. To fulfill these requirements, numerous DNA detection systems combined with elegant signal transduction and some amplification strategies based on the hybridization between a DNA probe and its complementary target have been described^26^,^27^,^28^,^29^,^30^,^31^,^32^. Recently, ECL technique has been developed to detect DNA. For example, Ru(bpy)_3_^2+^/TPA ECL system has been applied in the detection of sequence-specific DNA^33^. However, effective solutions for the above studies are limited, and this has driven the search for developing new ECL-based techniques in DNA detection. It was reported that DNA has the capacity to be intercalated with some small molecules into its grooves with high affinity. As a result, several DNA sensors have been developed by the use of the intercalation of small molecules probes into the DNA structures^34^,^35^,^36^,^37^,^38^. Ru(phen)_3_^2+^ (phen = 1,10-phenanthroline) and its derivatives have already been successfully intercalated into the grooves of DNA to develop ECL bioassays^39^,^40^,^41^,^42^.

Herein, we propose a novel strategy for highly sensitive detection of DNA based on Ru(bpy)_3_^2+^/QDs ECL system. QDs modified on the electrode can act as coreactant and generate strong anodic ECL with Ru(bpy)_3_^2+^ in neutral condition. Amino groups functionalized hairpin DNA can be bound to the carboxyl group of CdSe QDs via amide reaction. Ru(bpy)_3_^2+^ can be intercalated into the loop of hairpin through the electrostatic interaction, resulting in the strong ECL signal due to the reaction between Ru(bpy)_3_^2+^ and QDs. The loop of the hairpin DNA can be opened in the presence of target DNA, resulting in the release of Ru(bpy)_3_^2+^ and the decrease of ECL signal. The decreased ECL signal is linearly with the concentration of target DNA, which can be used in the sensitive detection of DNA.

## Results

### ECL of Ru(bpy)_3_^2+^ at CdSe QDs modified electrode

As reported, the strong anodic Ru(bpy)_3_^2+^ ECL can be observed in the presence of TPA. However, the limitation of TPA solution makes it necessary to explore new coreactants. Previous work revealed that QDs had good catalytic properties besides their good luminescent properties, which had rarely been studied in ECL^43^. Therefore, Ru(bpy)_3_^2+^ ECL was studied at the CdSe/GCE in the absence of TPA to explore the possibility of QDs as the coreactant.

High resolution transmission electron microscopy image displayed the crystalline feature of CdSe QDs with average size of about 3.7 nm (Figure S1). UV-vis absorption and fluorescence spectra of CdSe QDs were recorded and shown in Figure S2. CdSe QDs showed an absorption peak at 465 nm and a strong fluorescence emission at 565 nm, which was consistent with the previous work^44^.

The fabrication of QDs on a bare GCE was monitored by electrochemical impedance spectroscopy (EIS). Due to the existence of electrostatic repulse force between negatively charged QDs and [Fe(CN)_6_]^3−/4−^, the diameter of Nyquist circle increases with the amount of QDs modified on the electrode, which can indicate the assembly process of QDs. The EIS results of the GCE modified with different amount of CdSe QDs were recorded and shown in Figure S3A. It can be concluded that QDs are successfully modified on the GCE.

**Figure 1:**
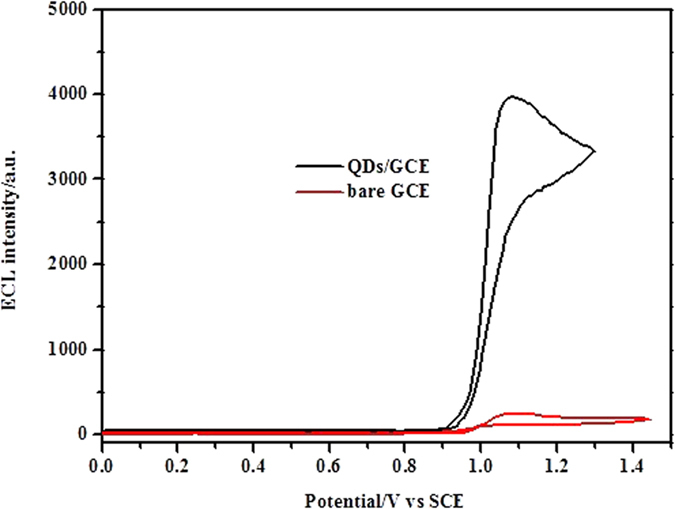
ECL curves of a bare GCE and a CdSe/GCE in Ru(bpy)_3_^2+^/PBS. Ru(bpy)_3_^2+^, 1.5 × 10^−4^ mol L^−1^; PBS, 0.1 mol L^−1^; pH, 7.4; scan rate, 100 mV s^−1^. If not mentioned additionally, all high voltages applied to the PMT were maintained at -800 V.

The ECL behaviors of Ru(bpy)_3_^2+^ were studied at a bare GCE and a CdSe QDs modified GCE (CdSe/GCE) in pH 7.4 PBS as shown in Fig. 1. In the absence of coreactant, the anodic ECL of Ru(bpy)_3_^2+^ is extremely weak. One strong anodic ECL peak can be observed at 1.10 V at the CdSe/GCE and the ECL intensity increases nearly 40 times compared with the bare GCE, indicating that CdSe QDs can act as the coreactant to react with Ru(bpy)_3_^2+^, resulting in the generation of a strong anodic ECL signal. Because carboxylated QDs are easier to immobilize DNA, the new ECL system can be used to fabricate ECL biosensor for the detection of DNA without adding other coreactant^45^. In order to further support the conclusion, several experiments were carried out as shown in Figure S4. Firstly, the same ECL behavior can be obtained at QDs modified gold electrode, revealing that strong ECL is result from QDs but not from electrode materials. Secondly, weak ECL signal is obtained at the bare GCE in Ru(bpy)_3_^2+^ solution, while strong ECL signal is obtained in QDs/Ru(bpy)_3_^2+^ mixing solution. Thirdly, the ECL intensity increased linearly with Ru(bpy)_3_^2+^ concentration but not QDs. These results can support sufficiently that QDs act as coreactant of Ru(bpy)_3_^2+^ ECL system.

The effects of the amount of QDs modified on the GCE on the ECL signals were studied and shown in Figure S3B. The ECL intensity increased with the amount of modified QDs. When the amount of QDs exceeded 20 μL, the increase of ECL signal leveled off. Because too thick QDs film is not facile for the electron transfer, 20 μL of QDs is chosen in the fabrication of ECL biosensor.

### Electrochemistry of Ru(bpy)_3_^2+^ at the CdSe/GCE

**Figure 2:**
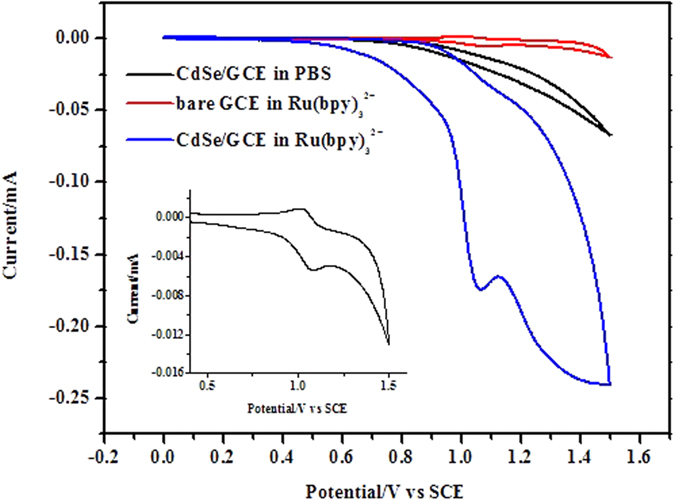
Cyclic voltammograms of a bare GCE and a CdSe/GCE in PBS with and without Ru(bpy)_3_^2+^. Ru(bpy)_3_^2+^, 1.5 × 10^−4^ mol L^−1^; PBS, 0.1 mol L^−1^; pH, 7.4; scan rate, 100 mV s^−1^. The inset is the enlarged CV of a bare GCE in PBS with Ru(bpy)_3_^2+^.

Cyclic voltammotric behaviors of Ru(bpy)_3_^2+^ on the bare GCE and the CdSe/GCE were studied in PBS (pH = 7.4) as shown in Fig. 2. At the bare GCE, a reversible redox peak observed at 1.10 V should be attributed to the oxidation-reduction of Ru(bpy)_3_^2+^. When the CdSe/GCE was studied in PBS without Ru(bpy)_3_^2+^, the QDs starts to oxidize at 0.70 V. This conclusion is also supported by the reported results^24^,^25^. When the CdSe/GCE was studied in PBS containing Ru(bpy)_3_^2+^, one apparent oxidation peak starts at 0.50 V and has the peak potential at 1.05 V, which could be assigned to the oxidation of Ru(bpy)_3_^2+^ because the peak current increased with the increase of Ru(bpy)_3_^2+^ concentration. The oxidation current of Ru(bpy)_3_^2+^ enhanced more than two orders of magnitude while the reduction peak nearly disappeared, suggesting that electrochemical reaction of Ru(bpy)_3_^2+^ could be catalyzed at the CdSe/GCE. Because the oxidation of QDs occurred at the same potential range, it is reasonable to deduce that the oxidation product of Ru(bpy)_3_^2+^ (Ru(bpy)_3_^3+^) can react with the oxidation product of QDs (QDs^+•^), resulting in the decrease of reduction peak of Ru(bpy)_3_^3+^. Meanwhile, the onset potential of the oxidation peak of Ru(bpy)_3_^2+^ at the CdSe/GCE located at ~0.50 V which is much less positive than that at the bare GCE (~0.85 V). The apparent increase in the oxidation current as well as the lower onset oxidation potential suggested that the QDs modified on the GCE exhibited good electrocatalytic effect on the oxidation of Ru(bpy)_3_^2+^.

**Figure 3:**
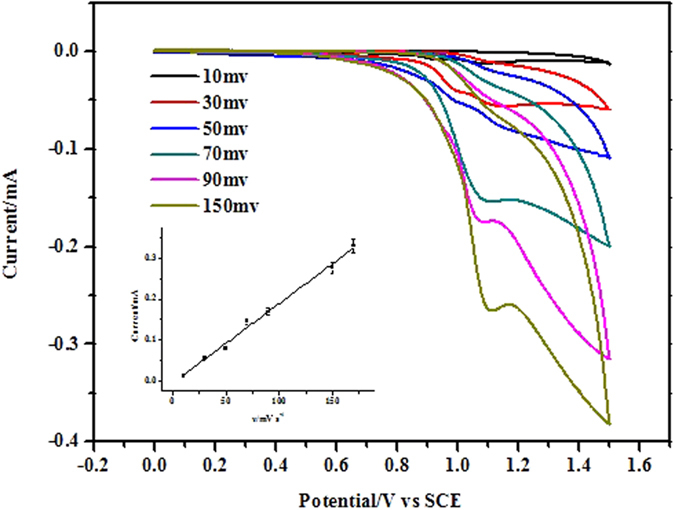
Effect of potential scan rate on the oxidation peak current of Ru(bpy)_3_^2+^ at the CdSe/GCE. Ru(bpy)_3_^2+^, 1.5 × 10^−4^ mol L^−1^; PBS, 0.1 mol L^−1^; pH, 7.4. The inset is the linear relationship between peak current of Ru(bpy)_3_^2+^ and potential scan rate.

The effect of potential scan rate on the oxidation peak of Ru(bpy)_3_^2+^ at the CdSe/GCE was studied as shown in Fig. 3. The oxidation current increased with the increase of potential scan rate. The peak current changed linearly with the potential scan rate, revealing that the oxidation process of Ru(bpy)_3_^2+^ on the CdSe/GCE is adsorption controlling process.

### Spectral investigation of the interaction between QDs and Ru(bpy)_3_^2+^

**Figure 4:**
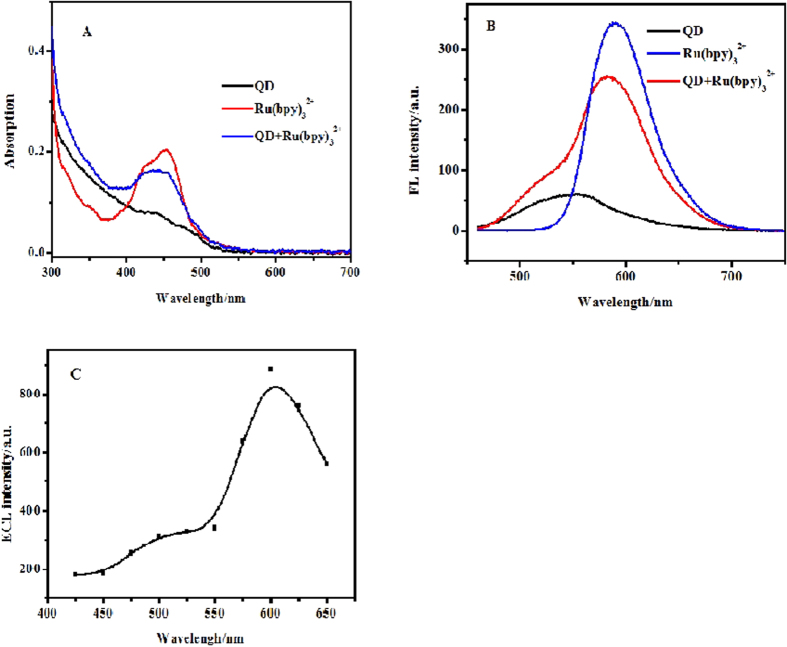
(**A**) UV-vis absorption spectra of QDs, Ru(bpy)_3_^2+^, and QDs+Ru(bpy)_3_^2+^. (**B**) Fluorescence spectra of QDs, Ru(bpy)_3_^2+^, and QDs+Ru(bpy)_3_^2+^. (**C**) ECL spectrum of Ru(bpy)_3_^2+^ at the CdSe/GCE.

UV-vis absorption, Fluorescence (FL), and ECL spectrum were recorded to explore the interaction between Ru(bpy)_3_^2+^ and QDs. In Fig. 4A, the mixture of QDs/Ru(bpy)_3_^2+^ exhibited a merging of absorption from separated QDs and Ru(bpy)_3_^2+^ in the range of 400–500 nm. Likewise, Ru(bpy)_3_^2+^ and QDs in the mixture also keep their FL properties as shown in Fig. 4B. These results suggested that no chemical reaction occurred between QDs and Ru(bpy)_3_^2+^. Thus, the light emission should be result from the reaction between the electrogenerated Ru(bpy)_3_^3+^ and QDs. There has no overlap between the absorption peak and the emission peak, revealing that energy transfer cannot occur between Ru(bpy)_3_^2+^ and QDs.

In Fig. 4C, the ECL spectrum included the maximum emission peak at 600 nm and a weak shoulder peak at 500 ~ 550 nm. The former agrees with the FL spectrum of Ru(bpy)_3_^2+^ while the latter agrees with the FL spectrum of QDs (Figure S2). The former ECL peak is more intense than the latter one, revealing that Ru(bpy)_3_^2+^ is the main emitter of the present ECL system while QDs can also emit weak light.

Therefore, the results from the spectral investigation suggested that Ru(bpy)_3_^2+^ in the mixture is the main emitter while QDs in the mixture can not only generate weak ECL but also act as coreactant to generate strong ECL with Ru(bpy)_3_^2+^.

### ECL mechanism of Ru(bpy)_3_^2+^ at the CdSe/GCE

It has been drawn from the ECL spectrum that the main luminophor of anodic ECL is Ru(bpy)_3_^2+^ while QDs can also generate weak emission. According to the ECL mechanism of Ru(bpy)_3_^2+^/TPA system as well as the electrochemical results, it is reasonable to propose that the electro-oxidation of Ru(bpy)_3_^2+^ to Ru(bpy)_3_^3+^ can be catalyzed by QDs. QDs can also be electro-oxidized at the same potential to generate strong reductive species QDs^+•^. Ru(bpy)_3_^3+^ can react with QDs^+•^ to generate excited state of Ru(bpy)_3_^2+^, which can emit light at ~600 nm. The mechanism of ECL process can be as follows:


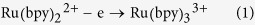



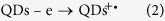



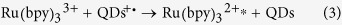



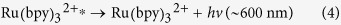


It was reported that the dissolved oxygen participated in the anodic QDs ECL in the species of O_2_^−•^, which could inject electrons into the hole injected QDs and generate anodic ECL^25^. Therefore, the extremely weak ECL peak in Fig. 4C should be generated as follows:


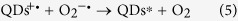



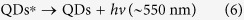


### Fabrication of ECL biosensor

Previous studies have revealed that Ru(phen)_3_^2+^ and its derivatives can be intercalated into the grooves of double-stranded DNA through electrostatic interaction, which can be used in ECL bioassay^39^,^40^,^41^,^42^. However, the amount of Ru(phen)_3_^2+^ that can be intercalated into the grooves of DNA is low, which limits the detection sensitivity. Therefore, in the present study, a hairpin DNA was used to integrate Ru(bpy)_3_^2+^ through electrostatic interaction. The interaction between Ru(bpy)_3_^2+^ and DNA was monitored by UV-vis and FL spectra as shown in Figure S5. FL spectra didn’t change while the UV-vis absorption peak at 250 nm changed when Ru(bpy)_3_^2+^ was intercalated into DNA, revealing that Ru(bpy)_3_^2+^ could be intercalated into DNA through the electrostatic interaction but not the chemical reaction.

**Figure 5:**
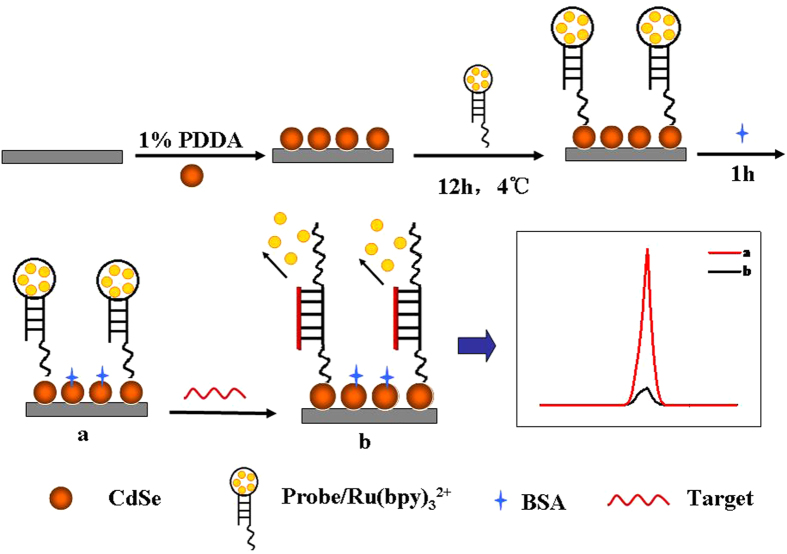
Schematic representation of the modification of the GCE and the detection of target DNA.

Figure 5 depicted the protocol of the proposed ECL biosensor based on the assembly strategy of DNA and QDs. First, QDs were modified on a bare GCE with the help of PDDA. Ru(bpy)_3_^2+^ was intercalated into the loop of hairpin DNA through electrostatic interaction to fabricate a probe. The probe could be immobilized on the QDs modified electrode through the interaction between –COOH groups of QDs and –NH_2_ groups of DNA. The strong ECL signal can be obtained by the reaction between Ru(bpy)_3_^2+^ and QDs. In the presence of the target DNA, the loop of hairpin DNA can be opened and the intercalated Ru(bpy)_3_^2+^ can be released from the modified electrode, which resulted in the decreased ECL signals.

**Figure 6:**
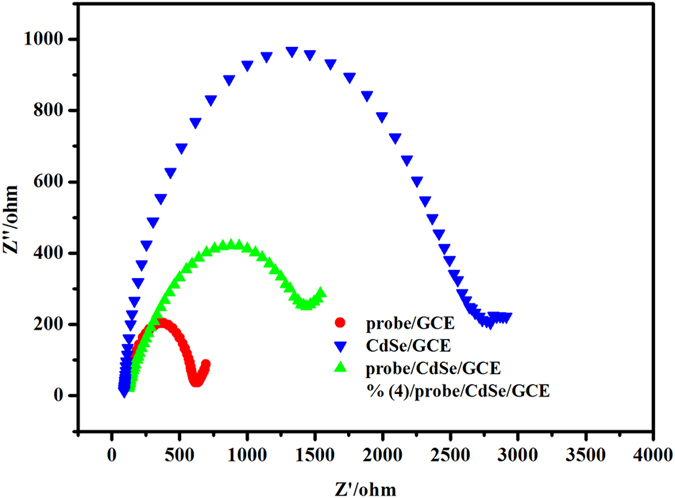
Nyquist diagrams of electrochemical impedance spectra recorded from 0.01 Hz to 10^5^ Hz for [Fe(CN)_6_]^3−/4−^ (10 mM, 1:1) in 10 mM pH 7.4 PBS containing 0.10 mol L^−1^ KCl at a bare GCE, a CdSe/GCE, a probe/CdSe/GCE, and a target/probe/CdSe/GCE.

As an effective tool for the characterization of the interface properties, electrochemical impedance spectroscopy (EIS) was used to monitor the biosensor fabrication process as shown in Fig. 6. The impedance spectra of all modified process consisted of a semicircle at high AC modulation frequency and line at low AC modulation frequency, demonstrating that the electrode process was controlled by electron transfer at high frequency and by diffusion at low frequency. The charge transfer resistance (R_ct_) which equals the diameter of semicircle reflects the restricted diffusion of the redox probe through the electrode surface. When QDs were modified on a bare GCE, R_ct_ increased greatly due to the electrostatic repulsive force between negatively charged QDs and [Fe(CN)_6_]^3−/4−^. The R_ct_ decreased when the Ru(bpy)_3_^2+^ intercalated probe DNA was connected on the QDs modified electrode due to the electrostatic attraction between the positively charged probe and [Fe(CN)_6_]^3−/4−^. When the target DNA was introduced, the loop of hairpin DNA was opened and Ru(bpy)_3_^2+^ was released from the probe. On the other hand, the insulating effect of DNA could perturb the interfacial charge transfer. As a result, the R_ct_ was greatly increased again. In order to confirm the above speculation, the Zeta potential results of QDs and DNA probe were recorded and shown in Figure S6. It can be found that QDs and probe DNA are negatively charged while Ru(bpy)_3_^2+^ intercalated probe DNA is positively charged, which can support our conclusion. These results suggested a successful stepwise fabrication of the proposed ECL biosensor.

**Figure 7:**
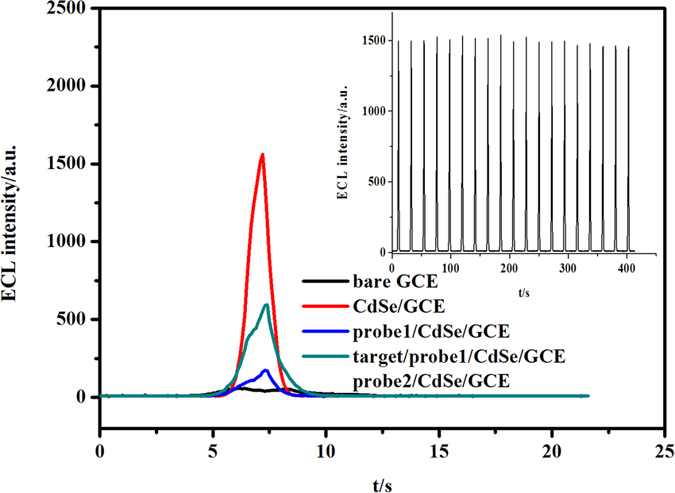
ECL responses of a bare GCE, a CdSe/GCE, a probe1/CdSe/GCE, a probe2/CdSe/GCE, and a target/probe1/CdSe/GCE in 0.1 mol L^−1^ PBS (pH 7.4). The inset is ECL emission from the probe1/CdSe/GCE under continuous cyclic voltammetry for 20 cycles.

The ECL responses of the modified electrodes in different stages were examined as shown in Fig. 7. Extremely weak ECL was observed at the CdSe/GCE, which is due to the anodic ECL of QDs between the dissolved oxygen. When the probe DNA (probe 1) was modified on the QDs, the intense ECL signal was obtained due to the ECL reaction between QDs and Ru(bpy)_3_^2+^. When the hairpin DNA with smaller loop was used as the probe (probe 2), the increased ECL signal is weaker than the hairpin DNA with larger loop, suggesting that Ru(bpy)_3_^2+^ could be intercalated into the loop of hairpin DNA. The ECL signal of the biosensor at 1.10 V remained at an almost constant value during 20 consecutive cyclic potential scanning as shown in the inset of Fig. 6. The stable ECL signals can facilitate the ECL sensor design. When the target DNA was hybridized with the hairpin DNA, the loop of hairpin was opened and the intercalated Ru(bpy)_3_^2+^ was released, resulting in the reduced ECL signal. The inhibiting effect of the target DNA on ECL signal of the biosensor can be used in the determination of DNA.

### Analytical performance of the biosensor

**Figure 8:**
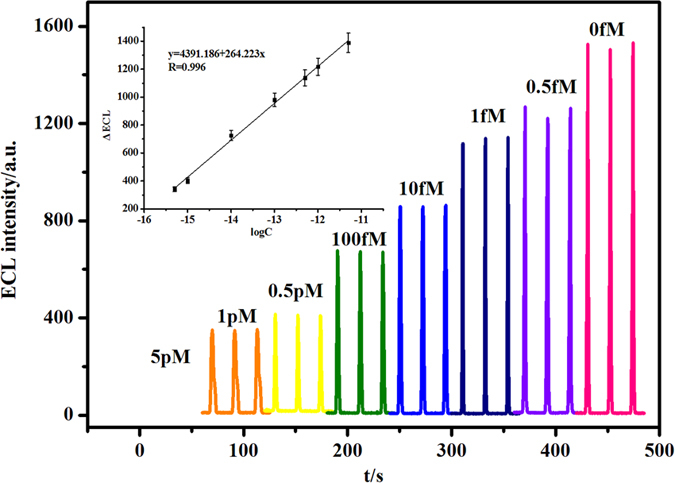
The ECL signals of the biosensor incubated with different concentrations of target DNA (0, 0.5 fM, 1 fM, 10 fM, 100 fM, 0.5 pM, 1 pM, 5 pM). The inset is the corresponding logarithmic calibration curve, ΔECL stands for the inhibited ECL signal of the modified electrode after the incubation in different concentration of target DNA.

The quantitative behavior of the fabricated ECL biosensor for DNA detection was assessed by measuring the dependence of the ΔECL upon the concentration of the target DNA under the optimized experimental conditions as shown in Fig. 8. With the increase of the concentration of the target DNA, the ECL of Ru(bpy)_3_^2+^ decreased as the result of opening the hairpin segment and releasing Ru(bpy)_3_^2+^. The ΔECL varied linearly with the logarithm of target DNA concentration over the range of 5.0 × 10^−16^–5.0 × 10^−12^ mol L^−1^ with the correlation coefficient of 0.997 as shown in the inset of Fig. 8. The detection limit for target DNA was estimated to be 1.9 × 10^−16^ mol L^−1^ (3σ). The comparison of the various ECL biosensors was listed in Table S1. It can be found that the present biosensor is superior to most of other reported ECL biosensors.

### Specificity of the biosensor

**Figure 9:**
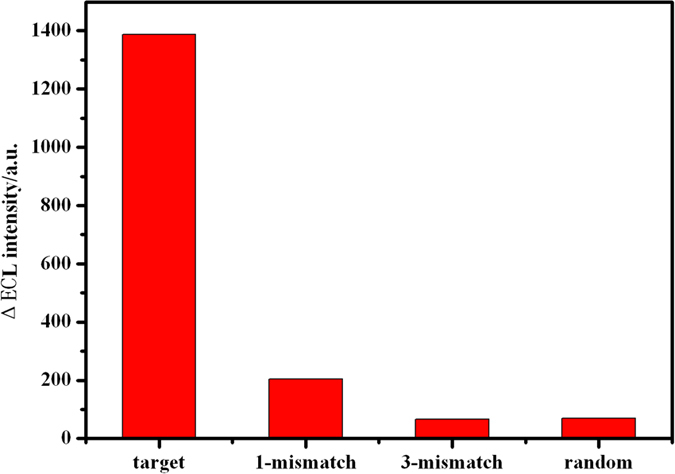
Comparison of the ECL intensity changes for the sensors hybridized with target DNA, one-base mismatched target DNA, three-base mismatched target DNA, and random sequence DNA in the same concentration (5 pM).

In order to investigate the specific response of the biosensor to DNA, control experiments were performed by incubating the biosensor in several aqueous solutions containing complementary (target) DNA, single-base mismatched DNA, three-base mismatched DNA, and noncomplementary (random) DNA, respectively. Figure 9 showed the comparison of the ECL intensity changes of the biosensors for the same concentration of different DNA. It can be found that a great ECL signal change (ΔECL) was observed after the ECL sensor was incubated in a complementary DNA, which was due to the loose of Ru(bpy)_3_^2+^ when the loop was opened. The ECL signal change for noncomplementary DNA was very small, attributed to the fact that the loop of hairpin DNA cannot be opened. The responses to the one-base mismatched DNA and the three-base mismatched DNA was only 15% and 5% of that for complementary target DNA. The comparison indicated that this method had high sequence specificity, and this detection approach had potential application in single nucleotide polymorphism analysis. The possible application of the biosensor in real samples detection was evaluated by recovery experiments determined in human serum samples. Two human serum samples were spiked with known concentrations of target DNA and were determined using the calibration curves of Fig. 8. The obtained recovery values range from 95 to 102%, demonstrating that the satisfactory results can be obtained in real samples (Table S2).

## Discussion

Taken together, CdSe quantum dots can catalyze the electrochemical oxidation of Ru(bpy)_3_^2+^ and strong anodic ECL signal can be observed at the CdSe QDs modified glassy carbon electrode in neutral condition, suggesting that CdSe QDs can act as novel coreactants for Ru(bpy)_3_^2+^ ECL system. This work has several key meritorious novelties. Firstly, CdSe QDs can act as coreactant of Ru(bpy)_3_^2+^ instead of TPA, which reveals new characters of QDs in ECL application. Secondly, the immobilization of QDs coreactant on the electrode can avoid the use of coreactant in solution and simplify ECL system. Thirdly, good biocompatibility of QDs is facile for the fabrication of ECL biosensor. Greatly improved sensitivity is realized via intercalation of more Ru(bpy)_3_^2+^ into the loop of hairpin DNA through the electrostatic interaction. The strong ECL between Ru(bpy)_3_^2+^ and QDs results in a sensitive ECL biosensor for DNA without signal amplification. The inherent selectivity of the probe endows the biosensor with high base discrimination ability. The proposed approach provides a promising detection platform for genetic analysis and clinic biomedical applications.

## Methods

### Chemicals and Apparatus

Ru(bpy)_3_Cl_2_, poly-(diallyldimethylammonium chloride) (PDDA, 20%, w/w in water, MW = 200000–350000), bovine serum albumin (BSA), N-hydroxysuccinimide (NHS), and 1-ethyl-3-(3-dimethylaminopropyl) carbodiimide hydrochloride (EDC), were purchased from Sigma-Aldrich. Other chemicals were analytical grade and double distilled water was used throughout. 3.0 × 10^−3^ mol L^−1^ stock solution of Ru(bpy)_3_^2+^ was prepared by dissolving Ru(bpy)_3_^2+^ in double distilled water. Working solution of Ru(bpy)_3_^2+^ was prepared by diluting stock solution with phosphate buffer solution (PBS). 0.1 mol L^−1^ pH 7.4 PBS was prepared by mixing the stock solutions of Na_2_HPO_4_ and NaH_2_PO_4_, and then adjusting the pH with 0.1 mol L^−1^ NaOH and H_3_PO_4_. Tris-HCl buffer (0.1 mol L^−1^, pH 7.4) was employed for preparation of DNA stock solution.

The DNA used in the work was synthesized and purified by Shanghai Sangon Biological Engineering Technology & Service Co., Ltd. (Shanghai, China). Two hairpin DNA probes with different diameter loop were adopted. The loop of probe-1 is complementary to the target DNA. The target DNA can hybrid to the loop of the hairpin DNA, which will cause the hairpin probe to adopt the open form^46^. The DNA sequences used are as follows:

Probe-1: 5′-NH_2_-(CH_2_)_6_-TTT TTT ***AAC TCC TTC TGC CCG TGT TT***G TAG GTG GAG TTC CC-3′ (the italicized part is the complementary strand of the target DNA)

Probe-2: 5′-NH_2_-(CH_2_)_6_-TTT TTT AAC TCC TTC GGA GTT CC-3′

Target DNA: 5′-AAA CAC GGG CAG AAG GAG TT-3′

1-base mismatched DNA: 5′-AAA C***T***C GGG CAG AAG GAG TT-3′

3-base mismatched DNA: 5′-AAA C***T***C GGG CAG ***C***AG GAG ***A***T-3′

Random DNA: 5′-GAG GGC CTG CAG GAT CAT TG-3′

The electrochemical measurements were recorded with CHI 660D electrochemical workstation (CH Instruments Co., China). The ECL emission measurements were conducted on a model MPI-M electrochemiluminescence analyzer (Xi’An Remax Electronic Science & Technology Co. Ltd., China) at room temperature, and the voltage of the photomultiplier tube (PMT) was set at -800 V in the process of detection. All experiments were carried out with a conventional three-electrode system, including a modified GCE as the working electrode, a platinum wire as the counter electrode and a saturated calomel electrode (SCE) as the reference electrode, respectively. A commercial 5 ml cylindroid glass cell was used as ECL cell and was placed directly in front of the PMT. High resolution transmission electron microscopy (HRTEM) was obtained by a JEOL-2100 transmission electron microscopy (JEOL, Japan). The UV-vis absorption spectra were obtained on a Shimadzu UV-3600 spectrophotometer (Shimadzu, Japan). The fluorescence measurements were carried out on a RF-5301PC FL spectrophotometer (Shimadzu, Japan). The ECL spectrum was obtained by collecting the ECL data at 1.10 V during cyclic potential sweep with 10 pieces of filter at 425, 450, 475, 500, 525, 550, 575, 600, 625, and 650 nm, respectively.

### Preparation of CdSe QDs modified electrodes

CdSe QDs were synthesized following the literature procedures^47^. The QDs were precipitated out with ethanol, centrifuged, dried under vacuum and kept in a refrigerator at 4 °C for further use. A glassy carbon electrode (3 mm in diameter) was mechanically polished with alumina pastes of 0.3 μm, and then cleaned thoroughly in an ultrasonic cleaner with alcohol and water sequentially. After it was dried with blowing N_2_, 20 μL of QDs solution was spread on the working electrode and dried at the room temperature to fabricate QDs modified GCE (denoted as CdSe/GCE).

### Preparation of ECL biosensor

The Ru(bpy)_3_^2+^ intercalated DNA probe (probe/Ru(bpy)_3_^2+^) was synthesized according to the literature^48^. 5 μM probe DNA and 1.5 × 10^−4^ mol L^−1^ Ru(bpy)_3_^2+^ were mixed at the volume ratio of 1:1 homogeneously, and kept at 4 °C for 15 h to intercalate Ru(bpy)_3_^2+^ into the loop segment of the probe DNA. 10 μL of 1% PDDA was dropped on the surface of the working electrode and dried in the air. Then 20 μL of CdSe QDs was spread on it and dried at room temperature. The modified electrode was immersed in pH 7.4 0.1 mol L^−1^ PBS containing 5 mM EDC and 10 mM NHS for 30 min to active the carboxylic group of the QDs. 10 μL of the probe/Ru(bpy)_3_^2+^ was deposited on the activated electrode and incubated overnight at 4 °C. The electrode was washed by tris-HCl buffer twice to remove the unbonded probe. Afterward, the electrode was immersed in 1% BSA solution for 1 h to block the nonspecific binding sites on the surface. The resulted electrode was incubated with target DNA with different concentrations for 2 h at 38 °C. The obtained electrode was washed twice with tris-HCl-EDTA (TE) buffer and used to measure the ECL response.

## Additional Information

**How to cite this article**: Dong, Y.-P. *et al.* Anodic Electrogenerated Chemiluminescence of Ru(bpy)_3_^2+^ with CdSe Quantum Dots as Coreactant and Its Application in Quantitative Detection of DNA. *Sci. Rep.* **5**, 15392; doi: 10.1038/srep15392 (2015).

## Supplementary Material

Supporting Information
