# Supplementary material for: Anodic Electrogenerated Chemiluminescence of Ru(bpy)32+ with CdSe Quantum Dots as Coreactant and Its Application in Quantitative Detection of DNA
Source: Sci Rep. 2015 Oct 16;5:15392. doi: 10.1038/srep15392 (PMC4607998; doi:10.1038/srep15392)
Supplement: Supporting Information [file srep15392-s1.doc]

Supporting Information

Anodic Electrogenerated Chemiluminescence of Ru(bpy)_3_^2+^ with Quantum Dots as Coreactant and Its Application to Quantitative Detection of DNA

Yong-Ping Dong^a,b^, Ting-Ting Gao^a,b^, Ying Zhou^a,b^, Li-Ping Jiang ^a,*^, Jun-Jie Zhu^a,*^


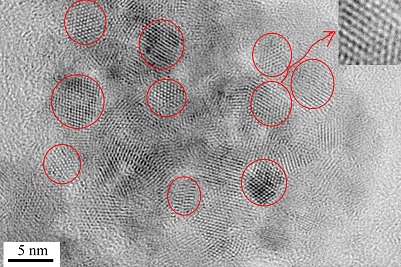


**Figure S1**. High resolution transmission electron microscopy image of CdSe QDs.

**Figure S2.** UV-vis absorption and fluorescence spectra of CdSe QDs.

**Figure S3.** (A) EIS of GCE modified with different amounts of CdSe QDs from 0.01 Hz to 10^5^ Hz for [Fe(CN)_6_]^3-/4-^ (10 mM, 1:1) in 0.1 mol L^-1^ pH 7.4 PBS containing 0.10 mol L^-1^ KCl. (a) bare GCE, (b) 5μL QDs, (c) 10μL QDs, (d) 15μL QDs, (e) 20μL QDs, (f) 25μL QDs. (B) Effects of the amount of the modified CdSe QDs on ECL signals.

**Figure S4.** ECL curves of bare GCE in QDs/Ru(bpy)_3_^2+^ mixing solution, QDs/GCE and QD/gold electrode (GE) in Ru(bpy)_3_^2+^ solution. The inset is the relationship between ECL intensities at the QDs/GCE and Ru(bpy)_3_^2+^ concentration.

**Figure S5.** FL spectra (A) and UV-vis absorption spectra (B) of Ru(bpy)_3_^2+^ and Ru(bpy)_3_^2+^+DNA

**Figure S6.** Zeta potential of Ru(bpy)_3_^2+^, probe DNA, Ru(bpy)_3_^2+^ intercalated probe DNA and QDs.

**Table S1.** Comparison of different ECL biosensors for the detection of DNA.

| ECL system | Linear range | LOD | Ref. |
| --- | --- | --- | --- |
| Ru(bpy)_3_^2+^/TPA | 0.1 pM-1.0 nM | 0.091 pM | 1 |
| Luminol/Pt NPs-CdS NCs/H_2_O_2_ | 5 fM -1.0 pM | 1.7 fM | 2 |
| CdS:Mn NCs/S_2_O_8_^2-^ | 50 aM-5 fM | 50 aM | 3 |
| CdTe@SiO_2_/ S_2_O_8_^2-^ | 0.1 nM-2 μM | 0.03 nM | 4 |
| Ru(bpy)_3_^2+^ doped Si NPs/TPA | 0.1 pM-2 nM | 0.05 pM | 5 |
| Ru(bpy)_3_^2+^/CdSe QDs | 0.5 fM-5.0 pM | 0.19 fM | This work |

1. X. F. Tang, D. Zhao, J. C. He, F. W. Li, J. X. Peng, M. N. Zhang, Anal. Chem. **2013**, 85, 1711-1718.

2. H. R. Zhang, J. J. Xu, H. Y. Chen, Anal. Chem. **2013**, 85, 5321-5325.

3. Y. Shan, J. J. Xu, H. Y. Chen, Chem. Commun. **2009**, 905-907.

4. W. Wei, J. Zhou, H. N. Li, L. H. Yin, Y. P. Pu, S. Q. Liu, Analyst, **2013**, 138, 3253-3258.

5. Q. X. Sun, G. Z. Zou, X. L. Zhang, Electroanalysis, **2011**, 11. 2693-2698.

**Table S2** Determination of target DNA added in human serum samples with the present ECL biosensor.

| Serum samples | Added target DNA/pM | Found target DNA/pM | Recovery/% | RSD/%, n= 3 |
| --- | --- | --- | --- | --- |
| Sample 1 | 1 | 0.95 | 95 | 3.3 |
|  | 5 | 5.08 | 100.2 | 3.1 |
| Sample 2 | 1 | 1.02 | 102 | 4.5 |
|  | 5 | 4.98 | 99.6 | 2.8 |
